# Respiratory-Related Hospitalizations following Prophylaxis in the Canadian Registry for Palivizumab (2005–2012) Compared to Other International Registries

**DOI:** 10.1155/2013/917068

**Published:** 2013-06-19

**Authors:** Bosco Paes, Ian Mitchell, Abby Li, Tetsuhiro Harimoto, Krista L. Lanctôt

**Affiliations:** ^1^Department of Pediatrics, McMaster University, Hamilton, ON, Canada L8S 4K1; ^2^McMaster Children's Hospital, 1280 Main Street West, Room HSC-3A, Hamilton, ON, Canada L8S 4K1; ^3^Department of Pediatrics, University of Calgary, Calgary, AB, Canada T3B 6A8; ^4^Medical Outcomes and Research in Economics (MORE) Research Group, Sunnybrook Health Sciences Centre, University of Toronto, Toronto, ON, Canada M4N 3M5

## Abstract

Respiratory syncytial virus (RSV) infection occurs commonly in infants aged ≤2 years, and severe infection results in hospitalization with accompanying morbidity and mortality. Palivizumab has been available for prophylaxis for the past 15 years. Prospective data on patients who received palivizumab from 2005 to 2012 has been assembled in the Canadian registry (CARESS) to document utilization, compliance, and health outcomes in both hospital and community settings. Long-term data is necessary to evaluate the impact of palivizumab on the incidence of RSV infections, minimize healthcare resources, and identify which infant subpopulations are receiving prophylaxis. A database search was also conducted for similar information from published registries, and hospitalization rates were compared to results from randomized clinical trials (RCTs).Overall hospitalization rates (percent; range) for respiratory-related illnesses and RSV-specific infection in infants who meet standard indications for prophylaxis were 6.6 (3.3–7.7) and 1.55 (0.3–2.06), respectively, in CARESS, which closely aligns with registry data from 4 other countries, despite the former comprising the largest cohort of complex patients internationally. Overall RSV-related hospitalization rates were lower across registries compared to equivalent patients in RCTs. Registry data provides valuable information regarding real-world experience with palivizumab, while facilitating the genesis of new research themes.

## 1. Introduction

Respiratory syncytial virus (RSV) continues to play a dominant role among the spectrum of viruses causing acute lower respiratory infection and subsequent hospitalization in infants and young children [1–6]. The burden of illness with accompanying morbidity, mortality, and associated healthcare costs is equally significant both within the community and world-wide [7–13].

Palivizumab, a humanized monoclonal antibody that targets the A antigenic site of the F-protein of RSV for the prevention of disease in high-risk children, demonstrates both neutralizing and fusion-inhibitory activity [14]. It was licensed in the USA by the Food and Drug Administration (FDA) in 1998 and subsequently by the European Medicines Evaluation Agency (EMEA) in 1999. Since its release, two major randomized, double-blind, placebo controlled trials [15, 16] and several follow-up studies [17, 18] have established the safety and efficacy of palivizumab in premature infants aged < 6 months who are ≤35 weeks gestational age and in children <2 years with hemodynamically significant congenital heart (HSCHD) or chronic lung disease (CLD).

Over several years, international registries have closely monitored patients who have received RSV prophylaxis, in order to determine utilization and compliance relative to country-specific or national pediatric guidelines and position statements [19–22]. The Canadian Registry for the evaluation of palivizumab (CARESS) was initiated in 2005, with the principal objective of documenting usage, compliance, and health outcomes of infants receiving RSV prophylaxis in both hospital and community settings during the annual RSV seasons. The registry tracks data on patient demographics, annual indications for prophylaxis, incidence of RSV infections, rates of hospitalization for respiratory-related and RSV-related illnesses with respective lengths of hospital stay, risk factors that govern time to hospitalization, acquired morbidities following hospital admission, and safety and compliance with palivizumab. 

The primary objective of this report is to document hospitalizations for respiratory illnesses (RIH) and RSV-specific infection (RSVH) within CARESS that spans the 2005–2012 RSV seasons and compare our results with published data from similar international registries and published randomized clinical trials (RCTs). 

## 2. Material and Methods

Infants who received at least one dose of palivizumab during any RSV season from 2005 to 2012 were eligible for inclusion in CARESS, if they had at least one of the following risk-factors: prematurity (≤35 completed weeks gestational age [GA]) without underlying medical disorders, CLD, HSCHD, or other “off-label” provincially approved medical conditions such as Down syndrome, congenital airway anomalies, immunodeficiency, or neuromuscular disorders. Preterm infants, 33–35 completed weeks GA, qualify for palivizumab only if they are considered at moderate (score 49–64) to high (score 65–100) risk for severe RSV infection and hospitalization based on a validated, Canadian risk-scoring model [23]. Children were excluded if a parent or legal guardian could not communicate in either English or French. Additionally, infants had to be recruited after their first injection of palivizumab and preferentially before receiving their third injection. 

 Subjects were enrolled by the local physician investigator and/or research nurse, which included providing the parent or legal guardian with an information package and consent form for review. Once consent was obtained, the research nurse completed an enrolment form to collect baseline data on patient demographics, prior medical history, neonatal course, and details of palivizumab administration. Following study initiation, the research nurse at the local site contacted the parent or legal guardian either in person or by telephone monthly, until the end of the RSV season, obtaining data on palivizumab administration, changes in baseline information, and specific facts regarding possible respiratory infections since the last contact. In the event of a hospitalization, and with parental consent, the relevant hospital records were reviewed by the site's research nurse for detailed information on patient diagnosis, reason for hospitalization, length of stay, days on respiratory support and/or intubation, and RSV specimen type and diagnostic test, as reported in the discharge summary. Collected data was logged into a central website. 

 Compliance was evaluated by two methods: actual number of doses prescribed versus expected number of doses for the duration of the RSV season and interdose interval. Palivizumab clinics currently administer about 5 monthly injections of palivizumab at 30 ± 5 day intervals, based on pharmacokinetic evidence from RCTs [15, 16, 24–26]. For expected number of doses, the number was calculated assuming monthly injections from the first dose to the end of the RSV season. The criterion for the start and end of the RSV season was defined by the previous study conducted by the Pediatric Investigators Collaborative Network on Infections in Canada [27]. For number of days between injections, 30 ± 5 day intervals were considered acceptable (i.e., as being within compliance). However, an interval of 20 ± 4 days between the first and second injections, likely results in higher trough levels after the first dose, offering better protection against the virus [28]. Therefore, an interval of 16–35 days between the first and second injections was considered compliant.

**Figure 1:**
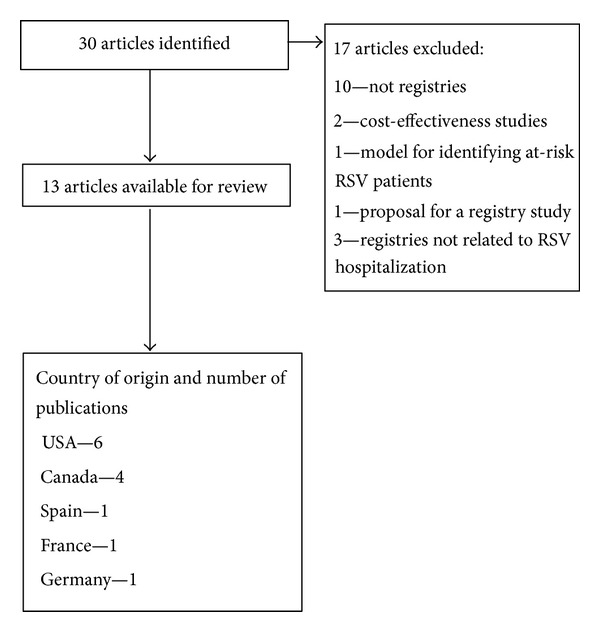
Flow chart of assembled articles from the scientific literature.

Comparative data from international registries was obtained through a search of Web of Science, PubMed, Medline, CINAHL, Cochrane, DARE, and OVID databases, using the key words “registry” AND “RSV” OR “respiratory syncytial virus” AND “newborn” OR “neonatal” OR “infant-newborn” AND “infant” AND “prophylaxis” OR “palivizumab”. All identified reports were compiled based on the aforementioned criteria and were further checked for references regarding additional pertinent studies, and a nucleus of key articles was derived for analyses (Figure 1).

## 3. Statistical Analysis

CARESS data were examined using standard descriptive methods. Data was entered into SPSS v20.0 (SPSS Inc, Chicago, Illinois) for analysis. The primary endpoint of this observational study was hospitalization. The RIH rate was defined as the number of children hospitalized for a respiratory-related illness/total number of children who received palivizumab. The RSVH rate was calculated using the formula: RIH × the number of RSV-positive children/the number of children with a respiratory illness tested. The characteristics of hospitalized versus nonhospitalized patients were evaluated to identify potential risk factors for respiratory illness-related hospitalization. The statistical tests used to determine these factors included Student's *t*-test and analysis of variance (ANOVA) for continuous variables and Pearson's chi-square (*χ* ^2^) test for nominal variables. An ANOVA was also applied in place of Student's *t-*test when more than two groups were assessed. A *P* value of <0.05 was considered statistically significant. 

To determine any factors that may affect time to RSVH, a Cox proportional hazards analysis was conducted using a backwards conditional method. Patients were followed for up to 30 days after their final injection. Results are presented in terms of hazard ratios (HR), with 95% confidence intervals (95% CI). 

## 4. Results

**Figure 2:**
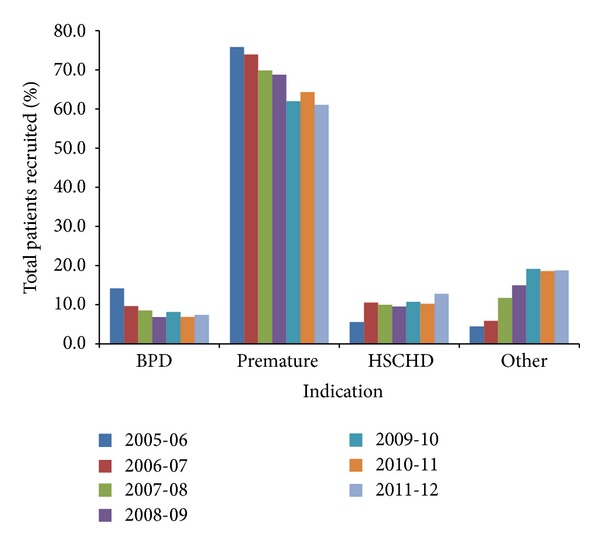
Palivizumab indications across seasons. Indications are subcategorized into chronic lung disease (BPD/CLD, *n* = 1048), premature (*n* = 8751), hemodynamically significant congenital heart disease (HSCHD, *n* = 1414), and “other” (*n* = 2097). The “other” group comprises infants with serious underlying medical disorders.

A total of 13,310 patients were recruited across 32 sites since the 2005-2006 RSV season. The proportions of patients recruited for the CARESS study are representative of the population proportions found by Statistics Canada in their latest quarterly demographic estimates [29]. Over the 7 years, 65.7% of patients prophylaxed with palivizumab were premature, 7.9% had CLD only and were not classified in any other category, 10.6% had HSCHD, and 15.8% had “other” serious medical disorders.  Figure 2 shows the distribution of patients based on indication for prophylaxis from 2005 to 2012.  Table 1 compares demographics across the indications. There were statistically significant differences between the groups in percent Caucasians, mean birth weight, enrolment and gestational age, daycare attendance, family history of atopy, multiple births, household smoking and more than 2 smokers in the home, siblings, siblings in daycare, and >5 people in the household. A *post hoc *analysis was conducted using the Tukey test to determine which indications contributed to the statistical significance. Birth weight across all indications was statistically significantly different from the “other” subcategory (*P* < 0.05). With regard to enrolment weight, the premature group was significantly different from the other 3 indications (CLD, HSCHD, and “other”; *P* < 0.005), while the HSCHD and “other” groups were similarly significantly different (*P* < 0.05). For gestational age, with the exception of the CLD and premature groups (*P* = 0.953), the indications were all significantly different from each other (*P* < 0.05). Over the seven RSV seasons encompassed by CARESS, there has been a 4.3-fold increase in the percentage of patients recruited that have been prophylaxed for serious underlying medical disorders, from 4.4% in the 2005-2006 RSV season to 18.8% in the 2011-2012 RSV season. Within the “other” category, there has also been a change in the distribution of recruitment in each subcategory (Table 2). More than >50% of the patients comprise the miscellaneous subcategory, which suggests that overall greater numbers of patients are receiving “off-label” palivizumab because of their illness severity.

More than 50% of the patients received respiratory support (59.4%) and oxygen therapy (52.6%) during the neonatal period. The average ± standard deviation duration of respiratory support was 23.3 ± 35.8 days, and the average duration of oxygen therapy was 37.5 ± 64.9 days. The average length of hospital stay after birth was 50.6 ± 80.8 days. Not surprisingly, significantly higher percentages of premature and CLD patients received respiratory support than HSCHD and “other” indications (63.8% and 76.1% versus 40.2% and 45.9%). Compared to HSCHD and “other” indications significantly higher percentages of subjects in the CLD group received oxygen therapy (84.7% versus 44.8–52.6%) and had documented necrotizing enterocolitis (6.4% versus 2.0–3.3%), sepsis (30.9% versus 8.9–14.8%), and surgery for patent ductus arteriosus (19.9% versus 3.6–6.6%). 

### 4.1. Palivizumab Utilization

Overall, patients received 98.2% ± 32.1% of their expected injections. Using inter-dose intervals, overall, 73.2% of infants were compliant. The 2006-07 season had a lower percentage of compliant subjects compared to other years (60.9% versus 67.8%–79.8%, *P* < 0.00005). 

### 4.2. Hospitalizations for Respiratory Illness-Related Events

**Figure 3:**
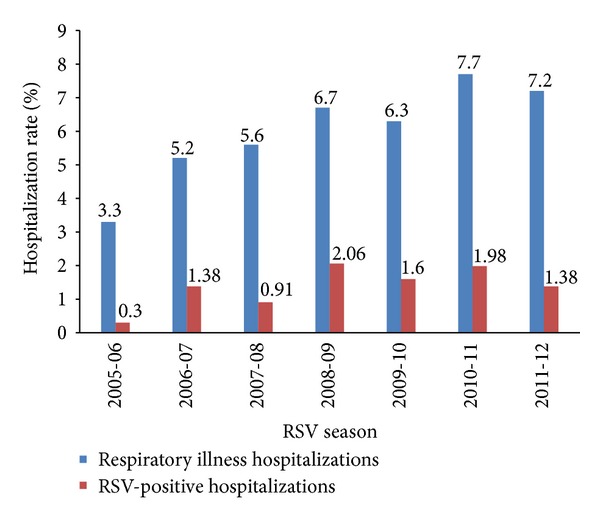
Hospitalizations for respiratory-related illness and RSV-positive infection (2005–2012).

Of the 13,310 patients that have been enrolled into the CARESS study, 875 patients had a total of 1,022 hospitalizations for a respiratory illness, giving a hospitalization rate of 6.6%. Patients were hospitalized for a range from 0 to 6 episodes per season. The average length of hospital stay was 8.8 ± 17.2 days with an average of 1.9 ± 8.9 days in intensive care. There may be an emerging trend towards higher hospitalization rates, with a low of 3.3% (2005-2006) and a high of 7.7% (2010-11) but with some variation (Figure 3).

Reviewing hospitalizations by indication (Table 3), a lower proportion of hospitalized versus nonhospitalized patients were premature (43.9% versus 67.3%, *P* < 0.0005) with a higher proportion ≤ 28 completed weeks GA (16.6% versus 14.5%). Conversely, there were a significantly higher proportion of hospitalized patients in each of the other indication groups than nonhospitalized patients apart from cystic fibrosis. However, no significant differences were found between hospitalized and nonhospitalized patients in terms of the proportion that had Down syndrome (*P* = 0.096), cystic fibrosis (*P* = 0.115), cardiac problems (*P* = 0.355) and various medical disorders (*P* = 0.188).

Comparing demographic information between hospitalized and nonhospitalized patients, a greater proportion attended daycare (5.8% versus 3.4%, *P* < 0.0005) and had siblings that attended daycare (26.2% versus 17.7%, *P* < 0.0005). Hospitalized infants also had more exposure to smoking, specifically, having a mother that smoked (19.1% versus 13.8%, *P* < 0.0005), a mother that smoked during pregnancy (18.4% versus 13.0%, *P* < 0.0005), smokers at home (32.2% versus 26.3%, *P* < 0.0005), and more than 2 smokers at home (13.6% versus 10.4%, *P* = 0.004). While a greater proportion of hospitalized infants had siblings (71.8% versus 60.9%, *P* < 0.0005), a lower proportion were from multiple births (20.8% versus 29.2%, *P* < 0.0005). A greater proportion of hospitalized infants also had a history of atopy in their immediate family (47.1% versus 40.0%, *P* < 0.0005).

Analyzing hospitalizations in terms of palivizumab compliance showed no significant difference between hospitalized and nonhospitalized patients in terms of compliance by expected number of injections (63.8% versus 66.2%, *P* = 0.149). However, based on interdose intervals, a significantly lower proportion of hospitalized patients were compliant with treatment (67.8% versus 73.6%, *P* < 0.0005). On average, hospitalized infants received a statistically greater number of injections than nonhospitalized infants (4.4 versus 4.2, *P* < 0.0005). There was no significant difference between the two groups in terms of the number of days between infants' first and second injections (28.8 versus 28.0 days, *P* = 0.149).

### 4.3. RSV-Related Hospitalizations

Of the 13,310 patients enrolled, 875 patients had a total of 1,022 RIHs. Among these, 847 RSV diagnostic tests were performed on 733 patients, predominantly using a nasal swab (30.6%) or a nasal aspirate (61.4%) and 28 (3.3%) were unreported. Of the 847 tests conducted, 177 (20.9%) were found to be positive in 173 patients. The RSV-positive hospitalization rate was 1.55% ([875/13310]×[173/733]).

On review of the 7 RSV seasons, the RSV-positive hospitalization rate (Figure 3) has fluctuated from 0.30% (2005-2006) to 2.06% (2008-2009). With regard to demographic data, a greater proportion of infants hospitalized with RSV infections had siblings (80.3% versus 61.3%, *P* < 0.0005), attended daycare (8.7% versus 3.5%, *P* = 0.001), and had siblings that attended daycare (32.4% versus 18.1%, *P* < 0.0005). A greater proportion also had a history of atopy in their immediate family (50.9% versus 40.4%, *P* = 0.006) were more likely to have been exposed to smoking, either by having a mother that smoked (20.2% versus 14.0%, *P* = 0.024), a mother that smoked during pregnancy (19.7% versus 13.3%, *P* = 0.021), or smokers in the household (37.6% versus 26.5%, *P* = 0.001). 

Table 4 shows the RIH and RSVH rates by indication with encountered morbidities during hospitalization. The RIH and RSVH rates ranged between 4.4%–12% and 1.36%–2.05%, respectively, across the groups and were statistically different (both *P* < 0.0005).

The average length of hospital and ICU (mean ± SD) stay for the total group was 8.8 ± 17.2 and 1.9 ± 8.9 days, respectively.

### 4.4. Cox Proportional Hazards Regression

**Figure 4:**
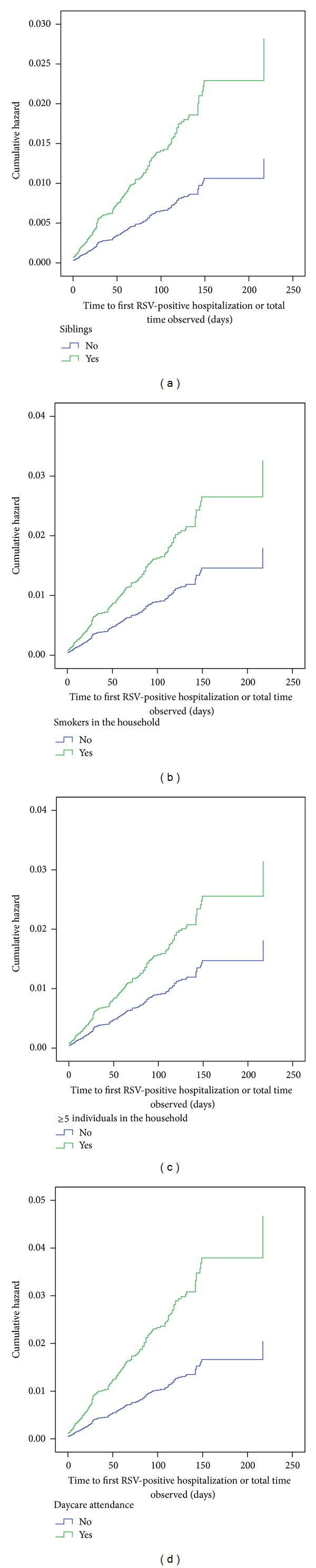
Hazard curves for the Cox proportional regression. (a) Comparing infants with siblings (green line) and those without (blue line). (b) Comparing infants with smokers in the household (green line) and those without (blue line). (c) Comparing infants with ≥5 individuals in the household (green line) and those with ≤5 members (blue line). (d) Comparing infants attending daycare (green line) versus nonattendees (blue line).

**Figure 5:**
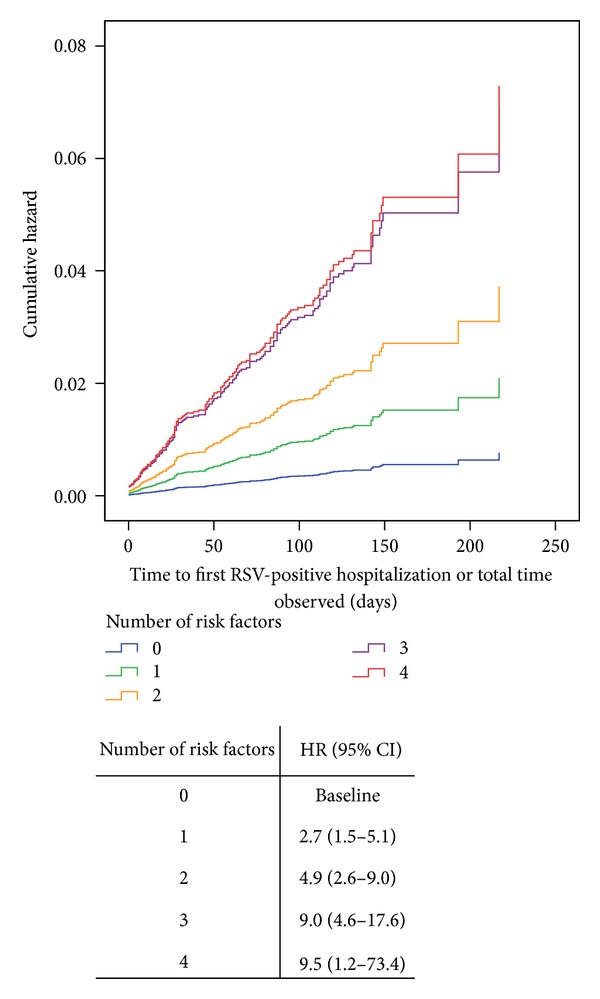
Hazard curves for the Cox proportional regression based on the number of risk factors per infant. Risk factors included were siblings, smokers in the household, ≥5 individuals in the household, and attending daycare.

To determine factors that may affect time to RSVH, a Cox proportional hazards regression was conducted. The overall model was significant (*χ* ^2^ = 65.847, df = 5, *P* < 0.0005) and showed that having siblings (Figure 4(a)) (HR = 2.1, 95% CI 1.4–3.3, *P* < 0.0005), smokers in the household (Figure 4(b)) (HR = 1.8, 95% CI 1.3–2.5, *P* < 0.0005), >5 individuals in the household (Figure 4(c)); HR = 1.7, 95% CI 1.3–2.4, *P* = 0.001), attending daycare (Figure 4(d)) (HR = 2.3, 95% CI 1.3–4.0, *P* = 0.004), and number of injections received (HR = 0.9, 95% CI 0.8–1.0, *P* = 0.032) were significant predictors of hospitalization. Other possible risk factors such as gestational age (*P* = 0.233), history of atopy (*P* = 0.081), gender (*P* = 0.776), being part of a multiple birth (*P* = 0.845), and compliance with treatment (*P* = 0.538) were not significant predictors. Interestingly, the hazard ratios also increased with increasing number of risk factors experienced by any infant (Figure 5). Infants with all 4 risk factors were 9.5 times more likely to be hospitalized with an RSV infection than those that had none of the risk factors.

Table 5 compares the data derived from international registries [28, 30–41], in 5 countries (USA, Canada, Germany, France, and Spain) accumulated from 2002 to 2012.  Table 6 outlines the RI and RSVH rates in various subpopulations of infants drawn from the respective registries versus the existing RCTs. Overall RIH rates for preterm infants <35 weeks GA and CLD patients ranged from 2.6% to 14.9% across studies while the corresponding RSVH rates inclusive of HSCHD were 0.2%–9.0%. In the RCTs, the RSVH rates for the same subgroups ranged from 1.8% to 7.9%. There was only one cystic fibrosis registry that found an adjusted HR for RSVH of 2.4 (95% CI; 0.8–6.6).

## 5. Discussion

Cumulatively, a total of 13,310 patients have been enrolled in the CARESS study, with 56.6% of the population being male, 70.4% Caucasian, and the majority were premature (≤35 completed weeks GA; 65.7%). The CARESS registry is the largest, comprehensive database of infants who have most currently received palivizumab (2005–2012) compared to other international registries that have published data from 1998 to 2007 (Table 5). Through the seven seasons of CARESS, there has been a steady increase in the percentage of patients that were given palivizumab prophylaxis for reasons that are not specifically indicated by Health Canada. This increase perhaps reflects emerging scientific data and an increased awareness of the potential morbidities and associated mortality with medical conditions such as neuromuscular disorders, Down syndrome, congenital airway and pulmonary abnormalities, immunocompromise, and cystic fibrosis [10–12, 42–49].

The 13,310 patients recruited into the CARESS study were given a total of 55,523 injections of palivizumab. More than half the patients received at least 4 injections per season, with an overall average of 4.2 ± 1.5 injections per infant. Compliance was 73.2% using interdose interval, with patients receiving 98.2% ± 32.1% of their expected injections. 875 patients were hospitalized a total of 1022 times for RIs within the CARESS registry, resulting in a hospitalization rate of 6.6%. Palivizumab was designed to preferentially target RSV subtypes A and B and reduce related hospitalizations. Singleton et al. [50] described the outcomes of 335 high-risk, Alaska Native palivizumab recipients from 1998 to 2001. RSV hospitalizations occurred in 20.6% (69/335), and 26.9% were admitted with respiratory illnesses during the same period, confirming the selective effect of palivizumab against RSV. Across the registries, the RIH rates for prophylaxed premature infants and those with CLD ranged from 2.6% to 14.9% [32, 34, 38, 41], the highest being in infants with CLD (10.5%–14.9%) [34, 41]. Paes et al. [31], previously documented that following RSV prophylaxis, infants with complex medical disorders when compared to a healthy cohort ≤35 weeks GA had an increased risk of RIH (HR = 2.0, 95% CI 1.5–2.5, *P* < 0.0005) but not RSVH. Moreover the RIH rates varied from 3.4% in infants with cystic fibrosis to 17.9% for those with neuromuscular impairments. This substantiates the fact that children with serious, underlying conditions remain prone to sever illness with a broad spectrum of viral infections apart from RSV. 

The cumulative CARESS (2005–2012) RSVH rate was 1.55%, and this is within the range of other palivizumab outcome registries (1.3%–8.1%) [28, 33, 37–39, 41]. The upper limit of 8.1% was found in the French registry [41], where the prevalence of CLD (81%) was significantly higher compared to the other described cohorts. In general, the registries reported lower RSVH rates compared to the RCTs; CLD (1.31%–5.8%) versus 7.9%, infants <32 weeks (1.5%–4.5%) versus 5.8%, infants 32–35 weeks (0.2%–1.6%) versus 2.0%, and HSCHD (1.99%) versus 5.3%, respectively. The US outcomes registry [37] documented steadily declining RSVH rates from 2000 (2.9%) to 2004 (0.7%) for all subjects, and 9.1% (*n* = 1, 123) had congenital airway anomalies or severe neuromuscular impairments. However, the CARESS database comprises 2,097 (15.8%) patients with a spectrum of serious underlying medical disorders who have received palivizumab, and these infants are likely to have higher breakthrough RSVH rates following immunization, despite optimal adherence to dosing schedules [15, 16, 31, 36]. Apart from striving to achieve 100% compliance, to further reduce RSVH, another potential strategy that can be adopted is a home-based prophylaxis program [36]. However, operationalizing this concept is administratively demanding and needs to be proven as cost-effective.

Infants with cystic fibrosis like patients with bronchopulmonary dysplasia (CLD) may develop severe, acute illness with RSV. In CF, synergy between virus and bacteria may lead to repetitive bacterial exacerbations [51]. While the majority of pediatric advisory bodies have universally approved RSV prophylaxis for CLD, there remains active debate as to whether CF patients merit palivizumab [43, 45, 52, 53]. The US CF foundation [54, 55] recommends that RSV prophylaxis should be considered for CF patients based on estimated net benefit which was graded as moderate and was derived from the limited number of existing uncontrolled studies. In the absence of a completed RCT in this population [42], the only available CF registry data [28] also suggests a potential benefit for prophylaxis, but more conclusive evidence from larger studies is awaited. 

The Cox proportional hazards analysis found that patients with siblings, those attending daycare, and who have either smokers or ≥5 people in their household were at higher risk of an RSV-positive hospitalization, with hazard ratios increasing concurrently with an incremental increase in the number of risk factors. These factors have a well-established association with severe RSV lower respiratory tract infection [56, 57], and similar findings were identified in several of the registries [33, 35–40]. Interestingly, in CARESS, compliance with treatment based on the expected number of injections rather than interdose intervals was not found to be a predictor of time to first RSV-positive hospitalization. This result is identical to what was found in the Palivizumab Outcomes Registry [37] suggesting that perhaps more stringent control of the timing of individual doses is perhaps more beneficial in reducing RSVH. The number of RSVHs is also likely dependent on the pharmacokinetics of palivizumab [15, 16, 26], with the highest frequency occurring between the first and second injection (range 31%–46%) with steadily declining rates to approximately 10% between the 3rd and 4th dose [28, 37].

There are perhaps several reasons that may account for the decreasing rates of RSVH seen in the Palivizumab Outcomes Registry [37] compared to the current CARESS study and older studies such as COMPOSS (1999-2000) [40], Romero (1998–2002) [38], and the IMpact-RSV trial (1996-1997) [15]. Since the Palivizumab Outcomes Registry, which spanned the 2000–2004 RSV seasons, encompasses data that is, more recent, the lower RSVH rate may reflect changes in the health system, such as preventative education initiatives targeted at patients, improved compliance, variability in RSV epidemiology, and hospital admission criteria. The fact that the CARESS registry did not show a similar trend in RSVH rates may be explained by the increasing percentages of patients being tested for RSV with more precise diagnostic tests such as polymerase chain reaction and the steady increase in the prophylaxis of patients with complex disorders who are more likely to be hospitalized with RSV.

There are several limitations of this data that deserve mentioning. Registries are handicapped by the absence of a control arm which would help to more clearly delineate the true impact of RSV prophylaxis as documented in the RCTs [15, 16]. Though the majority of assembled patients are similar because enrollment is founded on evidence-based local or national pediatric prophylaxis guidelines, variations do exist based on country-specific approval of populations such as 33–35 weeks GA infants and those patients with “off-label” medical, disorders for example, Down syndrome, neuromuscular impairments, and cystic fibrosis. However, the variance also facilitates new research endeavors especially in patients with complex medical disorders. RIH and RSVH detection rates are additionally influenced by the type of samples collected, the number and type of tests conducted, and the formula used for the standardization and reporting of results which were not always stated. Lastly, the changing demographic profile with varying levels of risk that contribute to RSV infection as in the Inuit population in the Canadian Arctic [58], term Alaska Native infants from the Yukon Delta [59], and the Aboriginal children in central Australia [60], combined with fluctuating epidemiological patterns of disease, may influence both RI and RSVH rates.

## 6. Conclusions

Over the past 15 years, palivizumab has been proven to be highly effective in decreasing RSVH rates, predominantly in children aged <2 years. The cumulative RIH and RSVH rates from 2005 to 2012 in the CARESS registry were 6.6% and 1.55%, respectively, and these incidences align closely with the data from 5 international registries across 13 publications in the scientific literature. Overall RSVH rates from the registries, which reflect everyday use of palivizumab in clinical practice for the key subpopulations of prematurity, CLD, and HSCHD, are lower than in the two randomized trials. The CARESS database also indicates that over the seven RSV seasons there is a growing trend to prophylax patients with other serious medical conditions from 4.4% in 2005-2006 to 18.8% in 2011-2012. This 4.3-fold increase indicates that pediatricians are strongly advocating for protection against serious RSV infection and possible sequelae in extremely high-risk patients. However, more evidence from well-conducted clinical trials is necessary before this strategy becomes standard of care for these infants.

## Figures and Tables

**Table 1:** Cumulative patient demographics by prophylaxis indication (2005–2012).

	Premature *N* = 8751	CLD *N* = 1048	HSCHD *N* = 1414	Other *N* = 2097	Total *N* = 13310	*P* value
Male, *n* (%)	4996 (57.1)	601 (57.3)	777 (55.0)	1161 (55.4)	7535 (56.6)	0.280
Caucasian, *n* (%)	6031 (68.9)	750 (71.6)	1047 (74.0)	1538 (73.3)	9366 (70.4)	<0.0005
Daycare attendance, *n* (%)	107 (1.2)	58 (5.5)	117 (8.3)	196 (9.3)	478 (3.6)	<0.0005
Atopy in the family, *n* (%)	3390 (38.7)	471 (44.9)	610 (43.1)	920 (43.9)	5391 (40.5)	<0.0005
Mean enrolment age (mo ± SD)	3.3 ± 3.1	10.3 ± 7.3	8.7 ± 7.3	10.0 ± 8.9	5.5 ± 6.2	<0.0005
Mean gestational age (wk ± SD)	30.9 ± 3.3	30.5 ± 5.8	38.1 ± 10.6	35.6 ± 5.7	32.4 ± 5.8	<0.0005
Mean birth weight (g ± SD)	1590 ± 632	1628 ± 1132	3048 ± 1154	2583 ± 1390	1902 ± 1043	<0.0005
Mean enrolment weight (g ± SD)	4017 ± 2167	7047 ± 2510	6092 ± 4423	7258 ± 3257	5082 ± 3087	<0.0005
Multiple births, *n* (%)	3140 (35.9)	250 (23.9)	89 (6.3)	340 (16.2)	3819 (28.7)	<0.0005
Mother that smokes, *n* (%)	1259 (14.4)	163 (15.6)	197 (13.9)	260 (12.4)	1879 (14.1)	0.067
Mother smoked during pregnancy, *n* (%)	1193 (13.6)	151 (14.4)	184 (13.0)	252 (12.0)	1780 (13.4)	0.188
Smoking in the household, *n* (%)	2471 (28.2)	276 (26.3)	335 (23.7)	465 (22.2)	3547 (26.6)	<0.0005
≥2 smokers in the home, *n* (%)	979 (11.2)	101 (9.6)	138 (9.8)	198 (9.4)	1416 (10.6)	0.042
Siblings, *n* (%)	5384 (61.5)	658 (62.8)	791 (55.9)	1364 (65.0)	8197 (61.6)	<0.0005
Siblings in daycare, *n* (%)	1588 (18.1)	153 (14.6)	242 (17.1)	451 (21.5)	2434 (18.3)	<0.0005
≥5 people in the household, *n* (%)	2509 (28.7)	238 (22.7)	299 (21.1)	505 (24.1)	3551 (26.7)	<0.0005

HSCHD: hemodynamically significant congenital heart disease; CLD: chronic lung disease.

**Table 2:** Changing patient profiles in the subcategory “other” over 6 RSV seasons*.

Indication, *n* (%)	RSV Season					
	2006-2007 *N* = 72	2007-2008 *N* = 169	2008-2009 *N* = 298	2009-2010 *N* = 462	2010-2011 *N* = 511	2011-2012 *N* = 538
Down syndrome	2 (2.8)	47 (27.8)	62 (20.8)	81 (17.5)	107 (20.9)	150 (27.9)
Cystic fibrosis	13 (18.1)	19 (11.2)	28 (9.4)	55 (11.9)	54 (10.6)	52 (9.7)
Congenital airway anomaly	16 (22.2)	46 (27.2)	50 (16.8)	66 (14.3)	91 (17.8)	75 (13.9)
Miscellaneous	41 (56.9)	57 (33.7)	158 (53)	260 (56.3)	259 (50.7)	261 (48.5)
Pulmonary disorders	13 (18.1)	12 (7.1)	65 (21.8)	90 (19.5)	89 (17.4)	73 (13.6)
Neuromuscular impairment	12 (16.7)	11 (6.5)	15 (5.0)	40 (8.7)	40 (7.8)	35 (6.5)
Immunocompromised	1 (1.4)	3 (1.8)	4 (1.3)	9 (1.9)	9 (1.8)	24 (4.5)
Cardiac disease ≥ 2 yr	0 (0.0)	7 (4.1)	7 (2.3)	7 (1.5)	10 (2.0)	11 (2.0)
Multisystem anomalies	5 (6.9)	6 (3.6)	32 (10.7)	15 (3.2)	30 (5.9)	41 (7.6)
Various medical disorders	10 (13.9)	18 (10.7)	35 (11.7)	99 (21.4)	81 (15.9)	77 (14.3)

*The 2005-2006 season was excluded as this information was not collected sequentially in all the subcategories for that year.

**Table 3:** Hospitalized versus nonhospitalized patients for respiratory-related illness based on indication.

Indication	Hospitalized (%) *N* = 875	Not hospitalized (%) *N* = 12435	*P* value (*χ* ^2^)
Premature	384 (43.9)	8367 (67.3)	<0.0005
≤28 weeks GA	145 (16.6)	1805 (14.5)	0.101
29–32 weeks GA	175 (20.0)	4645 (37.4)	<0.0005
33–35 weeks GA	64 (7.3)	1902 (15.3)	<0.0005
CLD	128 (14.6)	920 (7.4)	<0.0005
HSCHD	146 (16.7)	1268 (10.2)	<0.0005
Other	217 (24.8)	1880 (15.1)	<0.0005
Neuromuscular disorders	28 (3.2)	125 (1.0)	<0.0005
Airway anomaly	45 (5.1)	299 (2.4)	<0.0005
Cystic fibrosis	9 (1.0)	222 (1.8)	0.115
Down syndrome	38 (4.3)	411 (3.3)	0.096
Pulmonary	37 (4.2)	309 (2.5)	0.003
Cardiac ≥ 2 years	4 (0.5)	38 (0.3)	0.355
Immunocompromised	9 (1.0)	41 (0.3)	0.005
Multisystem anomalies	18 (2.1)	113 (0.9)	0.004
Various medical disorders	29 (3.3)	322 (2.6)	0.188

HSCHD: hemodynamically significant congenital heart disease; CLD: chronic lung disease.

**Table 4:** Respiratory illness (RIH) and RSV-related hospitalization (RSVH) rates and morbidities encountered during hospital stay according to indication.

	Prematurity	CLD	HSCHD	Other	*P* value
RIH					
RIH rate	4.4%	12.2%	10.3%	10.3%	<0.0005
Length of stay (mean ± SD)	7.9 ± 14.8	9.9 ± 25.0	8.7 ± 10.9	9.8 ± 18.3	0.469
Length of ICU stay (mean ± SD)	1.5 ± 4.8	1.7 ± 6.9	2.3 ± 5.7	2.5 ± 14.7	0.494
Days of intubation (mean ± SD)	0.7 ± 3.0	1.2 ± 6.6	0.9 ± 3.4	1.2 ± 8.9	0.643
Days of respiratory support (mean ± SD)	1.4 ± 4.8	2.4 ± 7.3	1.8 ± 5.2	3.1 ± 14.9	0.117
RSVH					
RSVH rate	1.36%	1.64%	2.05%	2.03%	<0.0005
Length of stay (mean ± SD)	7.7 ± 10.3	10.1 ± 11.4	7.2 ± 7.7	7.2 ± 5.8	0.776
Length of ICU stay (mean ± SD)	1.2 ± 2.6	3.1 ± 11.8	2.6 ± 5.0	1.6 ± 4.0	0.346
Days of intubation (mean ± SD)	0.6 ± 2.0	3.1 ± 11.8	1.6 ± 3.3	1.1 ± 3.3	0.180
Days of respiratory support (mean ± SD)	1.2 ± 2.7	3.6 ± 11.7	2.1 ± 4.7	2.1 ± 4.3	0.294

CLD: chronic lung disease; HSCHD: hemodynamically significant congenital heart disease; ICU: intensive care unit.

**Table 5:** Patient populations and outcomes of RSV prophylaxis across published registries.

Author/year/country	Study design	Characteristics of patients—*n*	Overall RSV hospitalization rate	Comments
Winterstein et al. [30]/2012/USA	National CF registry. Case control (1999–2006)	1,974 CF patients aged 0–2 yr over 2,875 patient seasons. Treated (PZ) compared to nontreated group	32 RSV-related hospitalizations. Adjusted HR for RSVH: 0.57 (95% CI; 0.2–1.6) Adjusted incidence rate: PZ, 2.4 (95% CI 0.8–6.6)	Low event rate. Only serious RSV illness was captured. Potential confounders

Paes et al. [31]/2012/Canada	CARESS registry-prospective, observational (2006–2010)	All treated (PZ). 4,880 infants ≤35 wk gestational age (GA) (group 1) compared to 952 with spectrum of medical disorders (MD; group 2)	RSVH: 1.3% versus 2.4% (*P* = 0.003) but ranged from 0.78% to 11.8% based on MD type. Hazard for RSVH was similar in both groups	Higher severity of illness in group 2. No control group. Study patients included only those approved for (PZ)

Paes et al. [32]/2012/Canada	CARESS registry-prospective, observational (2006–2011)	All treated (PZ). 5,183 ≤ 32 wk GA (group 1) versus 1,471 33–35 wk GA (group 2)	RSVH rates were similar (1.5% (Group 1) versus 1.4% (Group 2); *P* = 0.3). Hazard for RSVH was similar in both groups	No control group. Only moderate-high risk 33–35 wk infants were evaluated.

Mitchell et al. [34]/2011/Canada	CARESS registry-prospective, observational (2005–2009)	All treated (PZ). Total 5,286 infants: 3,741 (premature); 449 (CLD); 508 (HSCHD); 588 (other MD)	Overall RSVH was 1.38%: premature (1.12%); BPD (1.31%); HSCHD (1.99%); MD (2.78%)	No control group. Only patients approved for (PZ) were studied.

Simon et al. [33]/2011/Germany	German registry-observational (2002–2007)	All treated (PZ). 10,686 enrolled: evaluable patients 6,967 (<33 wk GA); 1500 (33–35 wk GA); 481 (>35 wk GA)	RSVH in worst-case scenario—2.5%	No control group. Not all patients were included and RSV tested. Possible missing data.

Frogel et al. [35]/2008/USA	Palivizumab outcomes registry-prospective, observational (2000–2004)	All treated (PZ). Total 19,548 infants, 19,474 with followup. PZ administered at home (1,226) versus clinic/office (17,641): 7,517 (<32 wk GA); 9,061 (32–35 wk GA); 2,285 (>35 wk GA)	RSVH: Home: 0.4%; clinic: 1.2%, *P* = 0.0139. Highest rate found in patients with mixed settings (5.8%)	No control group. Potential confounders were identified.

Cohen et al. [36]/2008/USA	Palivizumab outcomes registry-prospective, observational (2000–2004)	All treated (PZ). Total 19,548 infants. 1067 (acyanotic), 428 (cyanotic) CHD. 32.3% had HSCHD (485/1500). 468 (<32 wk GA); 327 (32–35 wk GA); 705 (>35 wk GA). Of these 448 also had CLD and 5 had CF.	Overall RSVH: 1.9% (all CHD); 1.6% (acyanotic); 2.6% (cyanotic)	No control group. 67.7% did not have HSCHD. Event rate possibly was underestimated.

Frogel et al. [37]/2008/USA	Palivizumab outcomes registry-prospective, observational (2000–2004)	All treated (PZ): Total 19,548 patients, 19,474 with follow-up. 7826 (<32 wk GA); 9,317 (32–35 wk GA); 2400 (>35 wk GA). Of these 4,349 (CLD); 1500 (CHD); 91 (CF)	Overall RSVH was 1.3%. <32 wk GA: 1.84; 32–35 wks GA: 0.83; >35 wk GA: 1.13. RSVH decreased in each subgroup from 2000 to 2004	No control group. Possible underestimation of event rate.

Parnes et al. [28]/2003/USA	Palivizumab outcomes registry-prospective, observational (2000-2001)	All treated (PZ). Total 2,116 infants: 986 (<32 wk GA); 957 (32–35 wk GA); 172 (>35 wk GA). Of these 500 (CLD); 102 (CHD); 12 (CF)	Overall RSVH: 2.9%. RSVH rate: 2.1% (prematurity without CLD); 4.3% (CHD); 5.8% (CLD)	No control group. 97% followup was achieved. 6% of infants >35 wk had no CLD

Romero [38]/2003/USA	Palivizumab outcomes registry-prospective, observational (1998–2002)	All treated (PZ). Total 4,669 infants (1998–2000) and 5,091 (2001-2002). Data for 2000-2001 [9]	Overall RSVH 2.3%; (1998-1999), 2.4% (1999-2000), 1.5% (2001-2002)	No control group

Pedraz et al. [39]/2003/Spain	Spanish registry (IRIS) case control (1998–2002)	Untreated (*n* = 1583; 1998–2000) versus treated (PZ; *n* = 1919; 2000–2002)	RSVH: control (13.25%); PZ (3.95%)	

Oh et al. [40]/2002/Canada	Canadian Therapeutic Products Program-prospective, observational (1999-2000)	All treated (PZ). Total 444/480 evaluable infants: Premature (345); CLD (40); both CLD and prematurity (68); others (27)	Overall RSVH (2.4%). RSVH in premature infants (1.6%); CLD (6.0%)	No control group. All patients were not tested for RSV and sampling method was not specified.

Lacaze-Masmonteil et al. [41]/2002/France	French Drug agency-prospective, observational study (1999-2000)	All treated (PZ). Total 516 infants (499 evaluable); 258 (<28 wk GA), 182 (29–32 wk GA), 31 (33–35 wk GA), 28 (>35 wk GA).	Overall RSVH (8.1%)	High proportion of infants had CLD (81%) which possibly influenced RSVH.

CF: cystic fibrosis; CHD: congenital heart disease; CLD: chronic lung disease; HR: hazard ratio; HSCHD: hemodynamically significant congenital heart disease; PZ: palivizumab; RSVH: respiratory syncytial virus-related hospitalization.

**Table 6:** RSV hospitalization rates within published registries based on specific subpopulations compared to published randomized clinical trials.

Author	Specific subpopulation	*RI hospitalization rate ^†^RSV hospitalization rate	Length of hospital stay in days	RCT	Comments
Parnes et al. [28] Winterstein et al. [30] Paes et al. [31]	Cystic fibrosis	^†^0.0% (*n* = 12) [28]; *26.2 and ^†^3.9/1000 season months [30]; *3.4% and ^†^1.14% (*n* = 117) [31]	Undefined [30, 31, 42]	At 6 months follow up RSVH (1.08% [1/92 PB]) [42]	Incomplete RCT [42]-PB (*n* = 92); placebo (*n* = 94)

Parnes et al. [28] Mitchell et al. [34] Frogel et al. [37] Romero [38] Pedraz et al. [39] Oh et al. [40] Lacaze-Masmonteil et al. [41]	Chronic lung disease (CLD)	^†^5.8% (*n* = 482) [28]; *10.5% and ^†^1.31% (*n* = 449) [34]; ^†^2.40% (*n* = 4,329) [37]; ^†^4.0% (*n* = NS) [38]; ^†^3.9% (*n* = NS) [38]; ^†^5.5% (*n* = 217, *P* < 0.007) [39]; ^†^3.3% (*n* = 35) [40]; *14.9% (*n* = 77); ^†^9.0% (*n* = 400) [41]	Undefined [15, 28, 34, 37–41] Overall total days/100 children for treated (PB) patients (≤35 wk GA + CLD): 36.4 (PB) versus 62.6 (Placebo) [15]	*n* = 762. RSVH: 7.9% (PB) versus 12.8% (placebo)-39% reduction [15]	Prevalence of CLD very high compared to other registries (81%) [41]

Parnes et al. [28] Paes et al. [32] Mitchell et al. [34] Frogel et al. [37] Romero [38] Pedraz et al. [39] Lacaze-Masmonteil et al. [41]	All Infants <32 wk GA [15, 38] Infants ≤32 wk without CLD [28, 32, 34, 37, 39, 41]	^†^4.5% (*n* = 949) [28]; *4.7% and ^†^1.5% (*n* = 5,183) [32]; ^†^1.84% (*n* = 7786) [37]; *10.5% and ^†^2.8% (*n* = 1,056) [38]; ^†^3.2% (*n* = 1,446) [38]; ^†^≤28 wk: (1.34%, *n* = 1,704) [34], ^†^5.4% (*n* = 739, *P* < 0.001) [39]; ^†^29–32 wk: 1.25%, (*n* = 1449) [34]; ^†^2.5% (*n* = 1170, *P* < 0.0000) [39]; ^†^7.72% (*n* = 440) [41]	Mean (±SD): 6.7 ± 5.4 [32]; <28 wk (6 ± 2.6) [38], 28–31 wk (14.8 ± 22.8) [38]; ≤32 wk (median 6 [IQR 4–9]) [39] Undefined [15, 28, 34, 37, 41]	*n* = 1,111. RSVH: 5.8% (PB) versus 11.0% (placebo)-47% reduction (*P* = 0.003) [15]	IMpact trial [15] Romero [38] reported on two US outcomes registry cohorts (1998-1999) and (1999-2000). The 2000-2001 cohort is reported by Parnes et al. [28]

Parnes et al. [28]	All infants without CLD [15]	^†^2.1% (*n* = 1,444) [28]	Undefined [15, 28]	*n* = 740. RSVH: 1.8% (PB) versus 8.1% (placebo)-78% reduction [15]	IMpact trial [15]

Frogel et al. [37] Romero [38]	Premature infants 32–35 wk GA [15, 37, 38]	^†^0.83% (*n* = 9294) [37];*2.6% (*n* = 1096) and ^†^1.5% (*n* = 548) [38]; ^†^1.3% (*n* = 972) [38]	Undefined [15, 37] Mean (±SD): 4.9 ± 3.6 [38]	*n* = 373. RSVH: 2.0% (PB) versus 9.8% (placebo)-80% reduction (*P* = 0.002) [15]	IMpact trial [15]

Paes et al. [32] Mitchell et al. [34] Frogel et al. [37]	Premature infants 32–35 wk GA without CLD [15, 28, 37] Infants 33–35 wk GA without CLD [32, 34]	^†^1.6% (*n* = 936) [28]; *3.7% and ^†^1.4% (*n* = 1,471) [32]; ^†^0.2% (*n* = 588) [34]; ^†^0.83% (*n* = 9,294) [37]	Mean (±SD): 5.2 ± 5.0 [32]; Undefined [34, 37]	*n* = 335. RSVH: 1.8% (PB) versus 10.0% (placebo)-82% reduction [15]	IMpact trial [15]

Parnes et al. [28] Frogel et al. [37]	Infants >35 wk GA	^†^0.6% (*n* = 164) [28]; ^†^1.13% (*n* = 2390) [37]	Undefined [15, 28, 37]		

Mitchell et al. [34]	Hemodynamically significant congenital heart disease (HSCHD) [16, 34]	HSCHD: ^†^1.99% (*n* = 508) [34]	Undefined [33] Total days/100 children: 57.4 (PB) versus 129.0 (Placebo; 56% reduction, *P* = 0.003) [16]	*n* = 1287: 682 (cyanotic), 605 (other). Overall RSVH: 5.3% (PB) versus 9.7% (placebo)-45% reduction. RSVH for cyanotic group: 5.6% (PB) versus 7.9% (placebo)-29% reduction (*P* = 0.285). RSVH for “acyanotic group”: 5.0% (PB) versus 11.8% (placebo)-58% reduction (*P* = 0.003) [16]	Feltes et al. [16]. All patients had hemodynamically significant CHD. Study was not powered for subgroup analyses. Incidence of serious adverse events was lower in the treatment arm: 55.4% (PB) versus 63.1% (placebo; *P* = 0.005)

CHD: congenital heart disease; CLD: chronic lung disease; GA: gestational age; HSCHD: hemodynamically significant congenital heart disease; NS: not specified; PB: palivizumab; RI: respiratory related hospitalization; RSVH: respiratory syncytial related hospitalization.
